# A Rare Case of Ascariasis-Induced Cholangitis Complicated With Klebsiella pneumoniae Bacteremia and Liver Microabscesses

**DOI:** 10.7759/cureus.12503

**Published:** 2021-01-05

**Authors:** Mohammad Aldiabat, Yasir Saeed, Donya Bani Hani, Sami Rabah, Bo Yu

**Affiliations:** 1 Internal Medicine, Lincoln Medical Center, New York, USA

**Keywords:** ascaris lumbricoides, cholangitis, microabscess, liver, bacteremia, klebsiella pneumoniae (kp)

## Abstract

Ascariasis, which is caused by *Ascaris lumbricoides,* is the most common gastrointestinal parasitic infection worldwide, with occasional invasion of the biliary tract leading to a variety of complications. In rare cases, pathogens carried on the surface of *A. lumbricoides* can complicate the course of the disease and lead to superimposed bacterial infections. In this article, we present a case of ascariasis-induced cholangitis complicated with *Klebsiella pneumoniae* bacteremia and multiple hepatic microabscesses. This article, which shows an association that was not reported in the literature before, aims to increase the awareness of clinicians for the possibility of the association between ascariasis and superimposed bacterial infection, specifically with *K. pneumoniae*.

## Introduction

Ascariasis, which is caused by *Ascaris lumbricoides,* is the most common gastrointestinal parasitic infection worldwide [1], with occasional invasion of the biliary tract leading to a variety of complications including cholecystitis, pyogenic cholangitis, and liver abscess. In rare cases, pathogens carried on the surface of *A. lumbricoides* can complicate the course of the disease and lead to superimposed bacterial infections. In this article, we present a case of a 17-year-old male who presented with ascariasis-induced cholangitis complicated with *Klebsiella pneumoniae* bacteremia and multiple hepatic microabscesses. To our knowledge, this association has not been reported in the literature before. The authors of this article aim to increase the awareness of clinicians for the possibility of the association between ascariasis and superimposed bacterial infection, specifically with *K. pneumoniae*.

## Case presentation

We present a case of a 17-year-old Hispanic male with a past medical history of Roux-en-y choledochojejunostomy, who presented to the hospital with a five-day history of fever, non-radiating right upper quadrant (RUQ) abdominal pain, nausea, vomiting, diarrhea, generalized weakness, confusion, and loss of appetite. The patient had watery, non-bilious, non-bloody vomiting and diarrhea, with no obvious foreign body passage. There was no history of skin discoloration or pruritus. The patient moved to the United States from Dominican Republic two years ago. Physical exam was remarkable for a young afebrile male with diaphoresis, restlessness, confusion, and RUQ tenderness. No abdominal distention or hepatomegaly was noted. Murphy, McBurney, and Rovsing signs were negative. The laboratory findings are reviewed in Table 1.

**Table 1:** Laboratory results. ALK PHOS, alkaline phosphatase; ALT, alanine aminotransferase; AST, aspartate aminotransferase; SGOT, serum glutamic oxaloacetic transaminase; SGPT, serum glutamic pyruvic transaminase; WBC, white blood cells

Test	First admission	Second admission	Reference
WBC	4.5	19.95	4.80-10.80 x 10^3^/mcL
Eosinophils count	0.1	0.04	0.10-0.40 x 10^3^/mcL
Eosinophils (%)	1.4	0.2	1.0-4.0
AST (SGOT)	23	84	≤40 U/L
ALT (SGPT)	25	59	≤41 U/L
ALK PHOS	103	125	40-130 U/L
Total bilirubin	1.24	2.12	0.20-1.20 mg/dL
Direct bilirubin	0.4	1	0.00-0.30 mg/dL
Albumin	4.1	4	3.5-5.2 g/dL
Lipase, serum	19	12	13-60 U/L
Amylase, serum	62	55	40-130 U/L
Procalcitonin	4.46	20.46	≤0.08 ng/mL
Blood cultures	Klebsiella pneumoniae	Klebsiella pneumoniae	Sterile
Hepatitis B surface antigen	Nonreactive	Nonreactive	Nonreactive
Hepatitis C antibodies	Nonreactive	Nonreactive	Nonreactive
Stool ova and parasite	Negative	Negative	Negative
Malaria and parasite screen	Negative	Negative	Negative
Blood parasites	Negative	Negative	Negative
Strongyloides antibodies	Negative	Negative	Negative
Clostridium difficile toxins A/B antigen, stool	Negative	Negative	Negative
Giardia antigen	Not detected	Not detected	Not detected
Entamoeba histolytica serology	Negative	Negative	Negative

**Figure 1:**
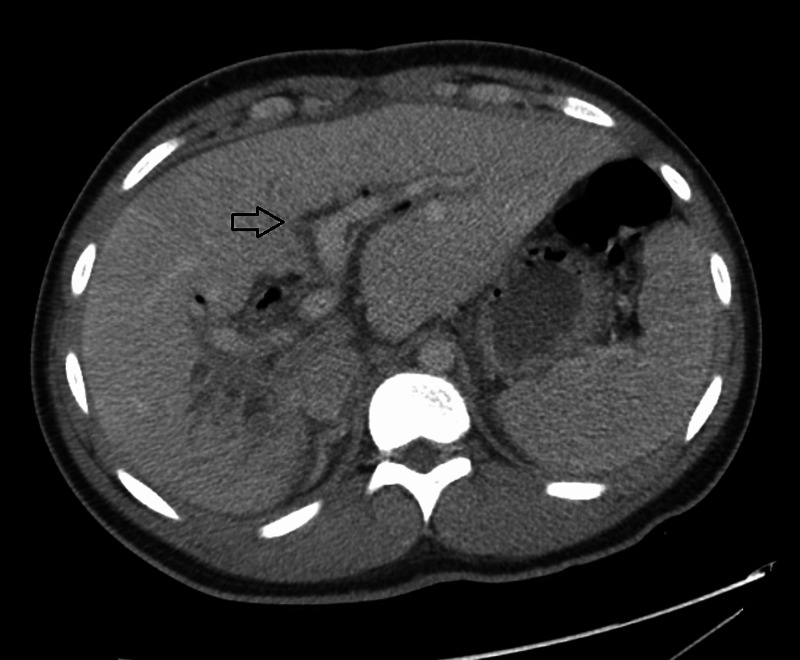
Abdominal CT scan showing multiple spheroidal foreign bodies within the dilated biliary tracts.

**Figure 2:**
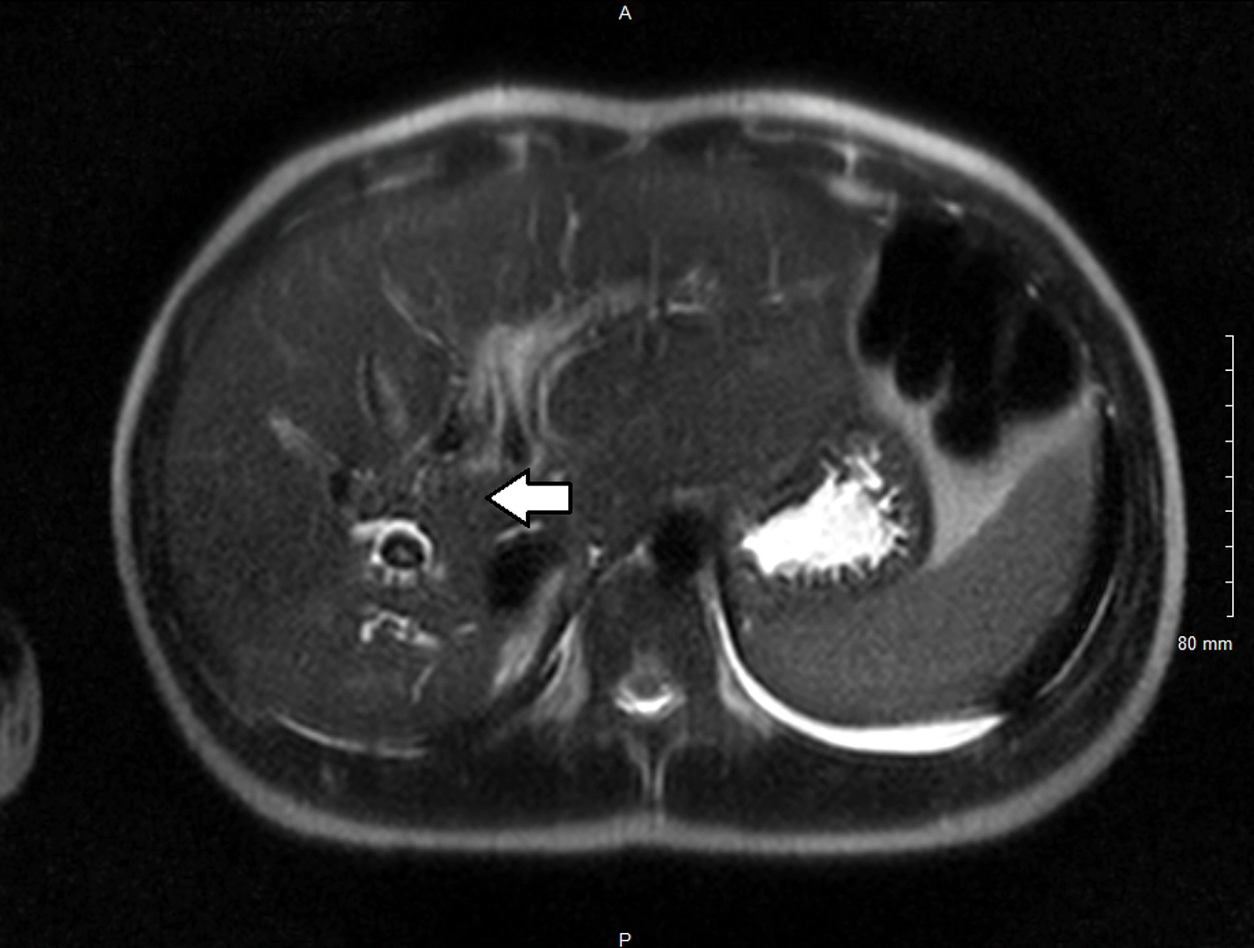
Magnetic resonance cholangiopancreatography confirmed findings of intrahepatic biliary dilatation in the right lobe of the liver with multiple curvilinear filling defects.

Abdominal CT scan (Figure 1) with contrast showed multiple spheroidal foreign bodies within dilated biliary tracts, enhancement of segment VIII and VII suggestive of inflammation, regional adenopathy, and pneumobilia within the biliary tree of the left lobe due to previous choledochojejunostomy. Magnetic resonance cholangiopancreatography (MRCP) (Figure 2) confirmed previous findings of intrahepatic biliary dilatation in the right lobe of liver with multiple curvilinear filling defects within the ducts with a 15-mm conglomeration centrally within the posterior segment of the right lobe, suspicious for parasitic infestation. Given the patient demographics and clinical and imaging findings, the patient was diagnosed with biliary parasitic infection (likely biliary ascariasis being the most common etiology) complicated with acute cholangitis and severe sepsis. The patient was admitted to pediatric intensive care unit for close monitoring and was started on broad-spectrum antibiotics. Stool testing was negative for ova and parasite, which can be explained by the absence of eggs secondary to an infection with a male *A. lumbricoides*. Blood cultures showed growth of pan-sensitive *K. pneumoniae*; therefore, antibiotics were adjusted to ceftriaxone IV, and the patient received praziquantel and albendazole to cover ascariasis and other parasitic infections. Following administration of anti-parasitic agents, the patient's clinical status improved significantly over his five-day stay, with resolution of symptoms. Repeated blood culture showed resolution of bacteremia. The patient was discharged with a seven-day course of cefdinir and nitazoxanide.

**Figure 3:**
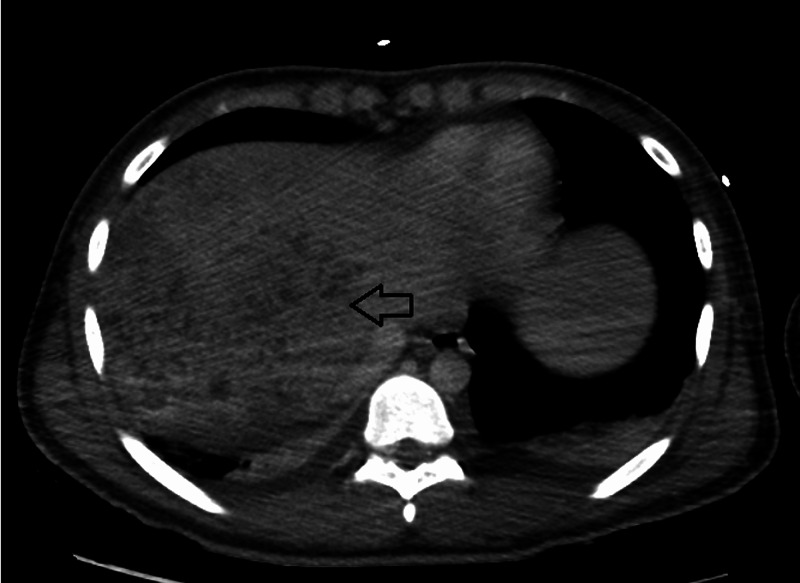
Repeat CT scan showed innumerable poorly defined foci of diminished attenuation in the right lobe of the liver representing multiple hepatic microabscesses.

**Figure 4:**
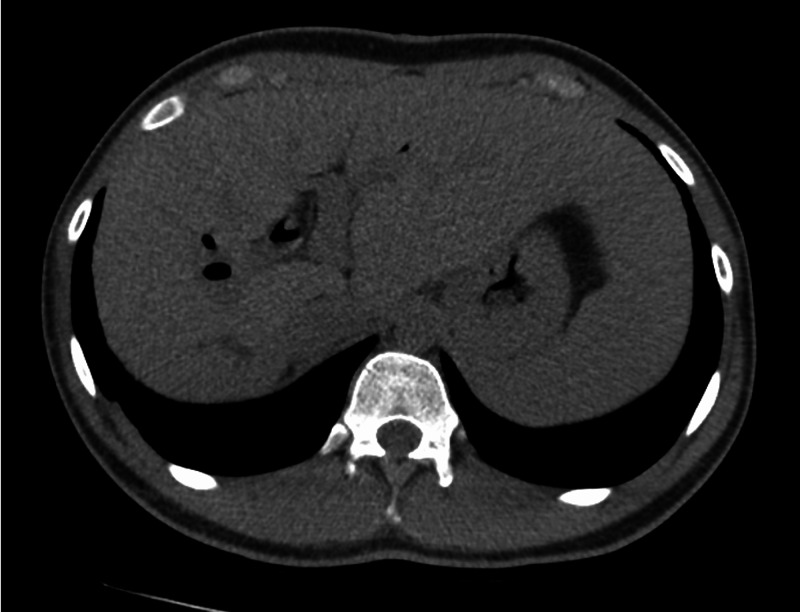
Significant improvement of intrahepatic biliary dilatation and resolution of the hepatic microabscesses after treatment.

Seven months later, the patient presented with similar complaints of fever, abdominal pain, vomiting, and diarrhea. Repeat laboratory testing is reviewed in Table 1. Blood cultures grew *K. pneumoniae*. Repeat CT scan (Figure 3) showed innumerable poorly defined foci of diminished attenuation in the right lobe of the liver, representing multiple hepatic microabscesses (that were poorly defined on previous scans), with persistent intrahepatic biliary ducts dilation representing reinfection of *A. lumbricoides*. Ova and parasite screen in addition to *Entamoeba histolytica* serology were negative. The patient received albendazole 400 mg orally, with clinical improvement and clearance of bacteremia after four days. On the seventh-week follow-up, repeat CT scan of the patient (Figure 4) showed significant improvement of the previous intrahepatic biliary dilatation and complete resolution of the hepatic microabscesses.

## Discussion

Ascariasis is the most common helminthic infection worldwide, with an estimated prevalence of 891 million around the world to be infected with the largest hookworm in the gastrointestinal tract [1]. The clinical scenario of intestinal ascariasis is usually benign, but in endemic areas such as India, it was found to be associated with one-third of patients with biliary and pancreatic diseases [2]. *Ascaris lumbricoides* can migrate to biliary duct through ampulla of Vater, where it can stay for up to three weeks and be complicated with biliary colic, biliary strictures, acalculous cholecystitis, ascending cholangitis, obstructive jaundice, and bile duct perforation with peritonitis before it migrates back again to the duodenum [3]. Rarely, penetration of liver parenchyma and colonization can happen in less than 1% of cases; thus, ascariasis-induced liver abscess should be suspected more in endemic areas of the world, such as Asia, Africa, and South America [4]. Treatment with benzimidazole (one dose of albendazole 400 mg) has a cure rate of 96.6% and an egg reduction rate of 99.9% [5], but reinfection can happen within six months secondary to persistent risk factors of poor sanitation and overcrowded houses [6], which may explain our patient's reinfection. In addition, anti-parasitic agents may not be fully effective against parasites present in the biliary tree, as these medications are poorly excreted in bile, and, therefore, surgical intervention may be required [7]. However, the patient presented in our case responded well to treatment with oral albendazole, and surgical intervention was not indicated.

Pyogenic liver abscess is a potential life-threatening situation, which is commonly caused by enteric Gram-negative bacilli such as *K. pneumoniae* and *Escherichia coli* [8]. Invasive liver abscess syndrome, an entity that describes liver abscess that occurs in the absence of hepatobiliary disease, is frequently reported in Asia (especially in Taiwan [9]), South Africa, and among Hispanic patients [10], hence our patient. In our case, the patient developed pyogenic cholangitis secondary to biliary ascariasis and was complicated with *Klebsiella* bacteremia and subsequent multiple hepatic microabscesses, an association, to our knowledge, that was not reported in the literature before. A retrospective study of 10 patients with ascariasis-induced liver abscess showed that blood cultures of all patients were sterile, whereas pus culture grew *E. coli* in four of them [11]. The presence of *A. lumbricoides* as a pathogen carrier of enteric *K. pneumoniae* explains the presence of bacteremia with the same pathogen during both admissions, which was complicated with multiple hepatic microabscesses on the second admission.

## Conclusions

Ascariasis is the most common helminthic infection worldwide, with biliary tree involvement happening in up to one-third of cases in endemic areas. Rarely, ascaris can serve as a carrier for enteric flora on its surface, which leads to secondary hepatobiliary infection and liver abscess formation. Ascariasis associated with liver abscess is a rare finding, and, prior to this report, its association with *Klebsiella* bacteremia has not been reported in the literature.
